# Risk factors and outcomes of surgical site infection after abdominoplasty: A retrospective study from Qatar

**DOI:** 10.5339/qmj.2026.29

**Published:** 2026-06-10

**Authors:** Humberto Guanche Garcell, Yamile Leon Rodriguez, Orlando Rafael Exposito Reyes, Georkis Martinez Garcia, Yulexi Hechavarria Jimenez, Yudileidy Creagh Coureaux, Evelin Noriega Campos, Osiris I. Escobar More, Ivis Lisset Leon Gutierrez, Yuliet Castillo Flores, Tania M. Fernandez Hernandez, Francisco Gutierrez García

**Affiliations:** 1 Infection Control Department, The Cuban Hospital, Hamad Medical Corporation, Doha, Qatar; 2 Surgical Department, The Cuban Hospital, Hamad Medical Corporation, Doha, Qatar; 3 Nursing Department, The Cuban Hospital, Hamad Medical Corporation, Doha, Qatar; 4 Quality and Patient Safety Department, The Cuban Hospital, Hamad Medical Corporation, Doha, Qatar; 5 National Institute of Nephrology, La Habana, Cuba

**Keywords:** Abdominoplasty, surgical wound infection, antibiotic prophylaxis, risk factors, retrospective studies, Qatar

## Abstract

**Background:**

Abdominoplasty, although classified as a clean elective procedure, carries a meaningful risk of surgical site infection (SSI), with reported SSI rates varying depending on patient factors and procedural complexity.

**Objective:**

To describe SSI incidence and associated risk factors after abdominoplasty at a Qatari tertiary facility, and to assess compliance with prophylaxis practices, antibiotic consumption, and costs.

**Methods:**

Retrospective cohort of all abdominoplasties at The Cuban Hospital, from January 1, 2022, to July 31, 2025 (N = 323). Data included demographics, comorbidities, perioperative glucose monitoring, procedure characteristics, and antibiotic use (prophylactic and therapeutic). The cohort comprised both simple abdominoplasties and combined procedures performed with additional interventions such as liposuction or hernia repair. SSI rates were analyzed using relative risk, 95% confidence intervals, and statistical tests with significance at P < 0.05.

**Results:**

A total of 323 patients underwent abdominoplasty with a mean age of 40.5 years (SD, 9.8; range, 19–63 years). The majority were female (276 patients, 85.4%), and 13.9% (45 patients) had a history of diabetes mellitus. Overall SSI incidence was 5.3% (17/323), highest in 2025 (8/112; 7.1%). Combined abdominoplasty had a higher SSI rate than simple procedures (10.4% [5/48] vs. 4.4% [12/275]; RR, 2.36 [95% CI, 0.88–6.35]). SSI was more frequent with diabetes (11.1% [5/45] vs. 4.3% [12/278]), overweight/obesity (7.3% [12/151]/4.3% [5/112] vs. 0% [0/42]), and higher postoperative glucose (mean: 7.97, standard deviation: 1.99; P = 0.04). Adherence to antibiotic prophylaxis was high for timing/selection/dose, but discontinuation beyond 24 hours and 7-day discharge prescriptions were common. Antibiotic consumption and costs were higher in SSI cases (355.9 vs. 280.5 DDD/100; 2807.6 vs. 2180 QR/100; P = 0.45; P = 0.72).

**Conclusion:**

SSI incidence after abdominoplasty was modest but increased with combined procedures and poorer postoperative glycemic control. Strengthening perioperative glucose protocols and enforcing 24-hour prophylaxis limits may reduce SSI and unnecessary antimicrobial use and costs.

## 1. INTRODUCTION

Abdominoplasty is a commonly performed body-contouring procedure intended to remove excess skin and subcutaneous tissue and restore abdominal wall integrity by plicating the rectus muscles. While generally classified as a clean elective surgery, abdominoplasty carries a non-negligible risk of postoperative complications, particularly surgical site infection (SSI). The reported incidence of SSI following abdominoplasty ranges from approximately 0.2% to over 32%, with higher rates observed in specific patient populations, such as those with elevated body mass index, comorbidities, or prior abdominal surgeries.^1,2^ In multicenter observational studies of plastic surgery, abdominoplasty ranks among the procedures with the highest infection rates, particularly when compared with breast or facial interventions.^1^

Multiple patient- and procedure-related risk factors have been associated with increased SSI rates after abdominoplasty. Independent predictors include obesity, diabetes mellitus, chronic obstructive pulmonary disease, smoking, prolonged operative duration, the use of surgical drains, and emergency or combined procedures.^1–3^ Furthermore, SSI risk may be exacerbated by factors such as previous abdominal surgeries and the extent of tissue undermining or concurrent liposuction. In a prospective cohort study, open abdominal approaches were associated with a 6.5-fold higher SSI risk compared to laparoscopic techniques, and emergent operations carried nearly a 5-fold increased risk. In addition to patient morbidity, SSIs contribute to prolonged hospital stays, increased health care costs, and the potential need for reoperation or long-term wound care.^4–6^

Antibiotic prophylaxis remains a cornerstone of SSI prevention in abdominal and plastic surgery. Current guidelines recommend the administration of a single preoperative dose of a first-generation cephalosporin (e.g., cefazolin) within 60 minutes before incision, particularly in clean-contaminated or high-risk clean procedures.^3^ In abdominoplasty, prophylactic antibiotics are frequently administered despite its classification as a clean procedure.^3–5^

In Qatar, abdominoplasty is commonly performed either as a standalone procedure or in combination with other surgical interventions, such as liposuction, hernia repair, or additional plastic surgery procedures.^7^ To date, no published data are available regarding abdominoplasty outcomes in Qatar. In 2025, a noticeable increase in the incidence of SSI following abdominoplasty was observed in our setting. This trend underscores the need to assess both the incidence and associated risk factors for SSI in this context, to inform evidence-based preventive strategies and improve postoperative outcomes.

## 2. METHODOLOGY

This retrospective cohort study was conducted at The Cuban Hospital, a member facility of Hamad Medical Corporation (HMC) in Doha, Qatar. The study period spanned from January 1, 2022, to July 31, 2025. All patients who underwent abdominoplasty procedures (323 procedures documented), either as single procedures or combined with other surgical procedures such as liposuction or hernia repair, were included.

### 2.1 Inclusion criteria

All elective abdominoplasty procedures were performed during the study period (January 2022–July 2025).

### 2.2 Exclusion criteria

Incomplete medical records, abdominoplasties performed for reconstructive purposes (e.g., following major trauma or oncologic resection), emergency procedures, and patients with severe uncontrolled comorbidities (e.g., decompensated cardiac, hepatic, or renal failure; American Society of Anesthesiologists (ASA) score > III).

### 2.3 Data collection

Data collection was performed retrospectively, extracted from electronic medical records using a study-specific paper recording form (study-specific data collection), designed to capture all relevant variables for each identified case. Recorded data were then transferred to a Microsoft Excel spreadsheet for cleaning, management, and statistical analysis. Variables collected included patient demographics, comorbidities, smoking habits, nutritional status on admission, surgical details (procedure duration, wound classification, ASA physical status score), and antibiotic prophylaxis (timing, selection, dose, discontinuation). Adequate compliance with the recommended timing of antibiotic prophylaxis was defined as administration within 60 minutes before surgical incision. The appropriate initial dose was 2 g of cefazolin, or 600 to 900 mg of clindamycin for patients with documented allergies, with an additional intraoperative dose of cefazolin administered 3 hours after the initial dose. Cefazolin or clindamycin prophylaxis was continued for 24 hours postoperatively; any extension beyond this period was considered inappropriate continuation, as was prescribing antibiotics at the time of discharge. Furthermore, antibiotic prescriptions during follow-up visits within 30 days after the procedure were recorded. Blood glucose measurements obtained during the preoperative clinic visit, at hospital admission, during the preoperative and postoperative periods (on the day of the procedure), and on the day following the surgical intervention were documented.

Compliance with preoperative chlorhexidine gluconate (CHG) showers and hair removal methods (clipping, shaving, laser, and depilation) was also assessed. Antibiotic consumption was quantified using Defined Daily Dose (DDD) per 100 patients in accordance with World Health Organization Guidelines for Anatomical Therapeutic Chemical (ATC) classification and DDD assignment,^6^ and related costs were calculated in Qatari Riyals (QR).

### 2.4 Outcome definitions

The primary outcome was the SSI incidence confirmed up to 30 days postoperatively, defined according to the Centers for Disease Control and Prevention and National Healthcare Safety Network (CDC/NHSN) criteria and HMC policies.^8–11^ Secondary outcomes included compliance rates with infection prevention practices (expressed as percentages per 100 procedures), measures of antibiotic consumption (DDD per 100 procedures), and cost (QR per 100 procedures).

### 2.5 Statistical analysis

To stratify procedural risk, based on the NHSN/CDC reference, a risk score was considered, with the 75th percentile duration of simple or combined abdominoplasty; those exceeding this duration were assigned one point.^12^ Additional points were assigned for contaminated wound classification and ASA score of III or above, generating a total risk score categorized into four groups (0, 1, 2, and 3). SSI incidence rates were compared across years using relative risk (RR) with 95% confidence intervals (CI). Data analysis was performed using SPSS, version 22.0. Categorical variables were compared with Fisher’s exact test, applying a significance level of P < 0.05.

### 2.6 Ethical approval

The study was approved by the Institutional Review Board and the Medical Research Center (MRC-01-25-1039; date: January 1, 2025), Doha, Qatar.

## 3. RESULTS

A total of 323 patients underwent abdominoplasty at the Cuban Hospital in Qatar between 2022 and July 2025. The mean age was 40.5 years (SD, 9.8; range, 19–63 years). The majority were female (276 patients, 85.4%), and 13.9% (45 patients) had a history of diabetes mellitus. In terms of nutritional status, most patients were overweight (163 patients; 50.6%) or obese (117 patients, 36.3%). Combined abdominoplasty was performed in 14.8% (48/323) of cases.

**Figure 1.**
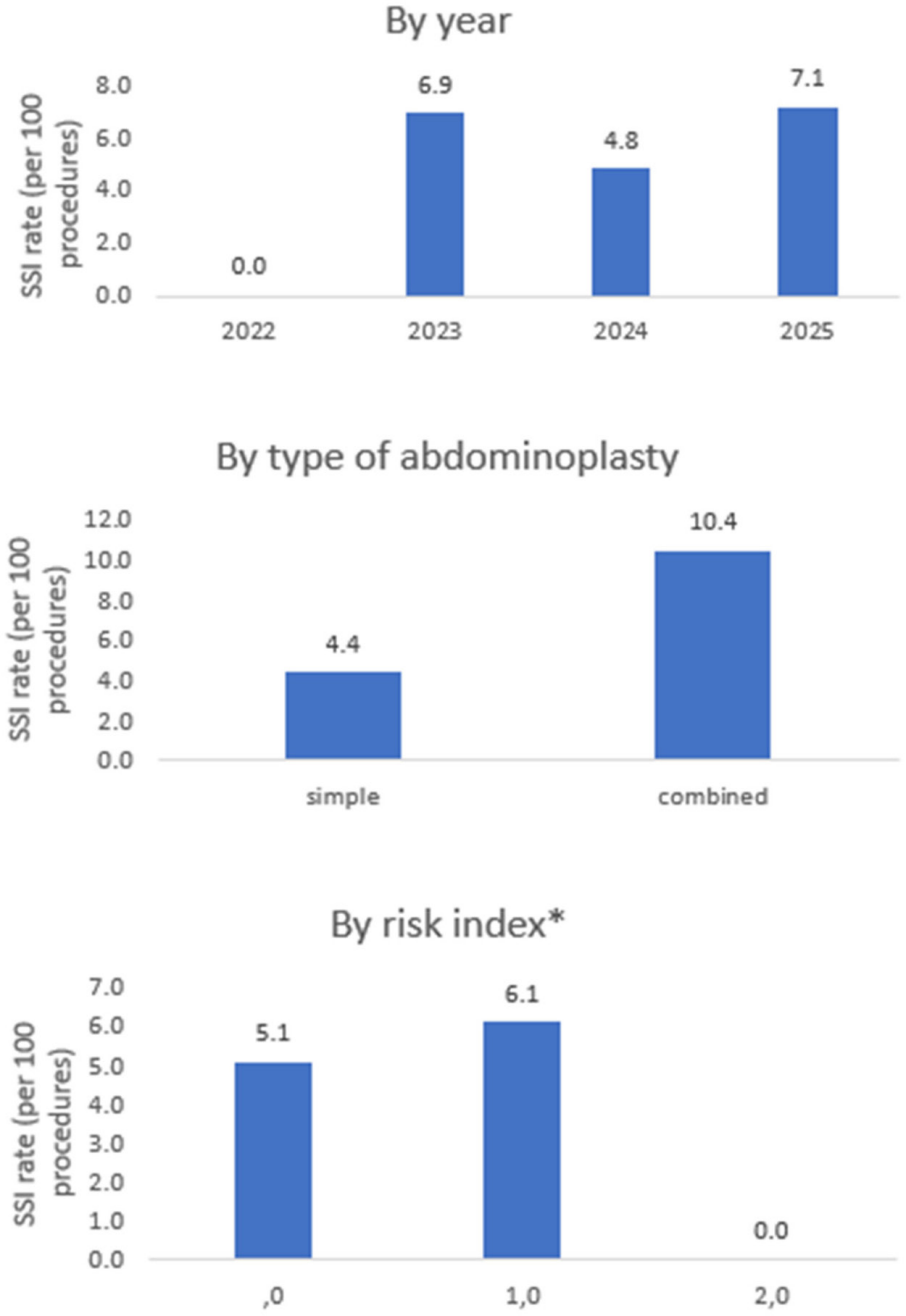
Surgical site infection rate in abdominoplasty according to year, procedure type, and risk index (per 100 procedures). *No surgical procedure with a risk index of 3 was performed.

Overall, 17 patients (5.3%) developed SSI. The SSI incidence following abdominoplasty varied across the examined factors. The overall SSI rate was highest in 2025 (8/112, 7.1%) (range 0.0%–7.1%). When categorized by procedure type, combined abdominoplasty demonstrated a significantly higher SSI rate of 10.4% (5/48) compared to 4.4% (12/275) for simple abdominoplasty, yielding a risk ratio (RR) of 2.36% (95% CI, 0.88–6.35), indicating a greater RR, though the confidence interval suggests considerable uncertainty. Analysis by the risk index showed a numerical increase in the SSI rate from 5.1% (12/236) in the risk index 0% to 6.1% in the RI 1 (5/82) (RR, 1.2 [95% CI, 0.43–3.33]). No SSI cases were reported in RI 2 (5/0). No surgical procedure was performed with RI 3 (Figure 1).

Potential risk factors are summarized in Table 1. Higher SSI rates were observed in patients with diabetes (11.1% vs. 4.3% in non-diabetics), in those who were overweight or obese (7.3% and 4.3%, respectively, vs. 0% in underweight/normal weight), and in patients undergoing combined procedures (10.6% vs. 4.3% for simple abdominoplasty). Likewise, SSI occurred more frequently in patients discharged with antibiotics (9.7% vs. 4.8%). Differences were also noted in mean postoperative glucose and in the interval between preoperative consultation and hospital admission; however, only postoperative glucose reached statistical significance (P = 0.04).

Among patients who did not develop an SSI, 47 (15.4%) received an antibiotic prescription within the first 30 days after discharge. In contrast, among patients who developed an SSI, 12 (70.5%) received antibiotics (P < 0.001). Notably, the antibiotic prescriptions issued to patients without evidence of infection were provided either by the primary surgeon or by other physicians, including those in emergency medicine or primary care. The documented reasons for these prescriptions included seromas, nonspecific inflammation, hematoma, or suspected cellulitis. Most treatments consisted of oral beta-lactam antibiotics.

Table 2 presents antibiotic consumption and cost during index admission, including discharge prescriptions, and compares patients who developed SSI with those who did not; prescriptions issued during postoperative clinic visits were not included. The expected consumption rate of cefazolin, when prescribed appropriately, is 125 DDD/100 procedures if the patient does not require a booster dose, and 200 DDD/100 procedures if a booster dose is needed. The corresponding costs are 1510 QR/100 procedures and 1812 QR/100 procedures, respectively. If clindamycin is administered instead, the expected consumption is 200 or 300 DDD/100 procedures, depending on whether the administered dose is 0.6 g or 0.9 g. The associated costs are 20 or 29 QR/100 procedures. In both cases, doses administered within 24 hours after the procedure are included, in accordance with policy. The most common non-compliant prescribing practices include extending antimicrobial therapy beyond 24 hours after the procedure (typically 48–72 hours) and prescribing a 7-day course of oral antibiotics (oral beta-lactams or clindamycin) at discharge. Although there were no statistically significant differences in either consumption or cost according to SSI status, antibiotic consumption was 26.9% higher in patients with SSI (355.9 vs. 280.5 DDD per 100 procedures; P = 0.45). Similarly, antibiotic costs were 28.7% higher among patients with SSI (2807.6 vs. 2180 QAR per 100 patients; P = 0.72).

## 4. DISCUSSION

In this study of 323 patients undergoing abdominoplasty, the overall SSI incidence was 5.3%. The risk was higher in combined abdominoplasty (10.4%) compared to simple abdominoplasties (4.4 %). Additional analyses showed elevated SSI rates in patients with diabetes mellitus, in those who were overweight or obese, and in individuals with higher postoperative glucose levels.

The incidence of SSI following abdominoplasty varies in the published literature, with figures ranging from 0.2% to 32%, depending on multiple factors. Likewise, the complexity and duration of the surgical procedure are key determinants in the risk of developing SSI.^11,13–17^ It is noteworthy that the risk of infection in combined abdominoplasties is approximately twice that of simple abdominoplasties. The incidence reported in our study may be considered low compared with other reports, with higher rates observed in combined abdominoplasties; however, the limited number of patients restricts comparability to other reports, and no defined trend toward a reduction in incidence was observed during the study period. Similarly, the definition of acceptable surgical procedure duration, established at the 75th percentile according to CDC/NHSN procedures, is limited by the number of cases, although it may be used as an initial approach to assess infection risk.

Analysis of risk factors did not reveal significant associations, except for postoperative glycemic control, which showed higher glucose levels in patients with SSI.^14,18^ Samuel et al. and Kantar et al. have identified diabetes mellitus and perioperative glycemic control as factors associated with infection risk. Consequently, it is recommended that postoperative blood glucose be monitored and controlled in all patients, regardless of a prior history of diabetes.^7^ Similarly, obesity has been associated with an increased risk of infection in abdominoplasty and other abdominal surgeries.^14,18–20^ Although no statistical association was demonstrated in the cases studied, it is noteworthy that the identified cases occurred among patients in the overweight and obese groups.

The appropriate use of antibiotic prophylaxis remains a cornerstone in preventing SSI.^8^ In our cohort, adherence was high regarding timing, antibiotic selection, and dosage; however, discontinuation practices were less consistent. The judicious use of antibiotic prophylaxis remains a cornerstone of SSI prevention. Prolongation of prophylactic antibiotic administration beyond the recommended duration has not demonstrated additional benefit in reducing SSI rates and instead promotes the emergence of antimicrobial resistance and undermines the overall efficiency of surgical care.^21^ Previous reports have demonstrated that institutional policies can improve compliance, yet our findings suggest challenges in sustaining these practices, particularly when new professionals with differing approaches join the team.^22^ This variability underscores the need for continuous education and reinforcement of standardized protocols. Furthermore, the impact on healthcare costs and antibiotic consumption cannot be overlooked, especially when antibiotics are used without evidence of infection—a practice previously documented in plastic surgery procedures.^4,5,23^ Such unnecessary use not only increases costs but also contributes to antimicrobial resistance, highlighting the importance of stewardship programs and monitoring adherence over time.

### 4.1 Limitations

This study has several limitations that should be considered. First, it was conducted in a single center with a relatively small sample size, which limits the generalizability of the findings; expanding the number of cases or performing multicenter studies could help address this issue. Second, data were collected retrospectively from medical records, which may be incomplete or subject to documentation bias, particularly for variables such as hair removal practices and smoking habits. Future research should aim to overcome these limitations through prospective, multicenter designs, standardized data collection, and inclusion of broader clinical and behavioral variables.

### 4.2 Recommendations

We recommend implementing standardized perioperative glycemic control protocols for all patients, regardless of diabetes status, and reinforcing institutional antibiotic policies to ensure sustainability and minimize unnecessary use. Also, limiting antibiotic prophylaxis to no more than 24 hours to reduce unnecessary antimicrobial exposure and the risk of resistance, and systematically addressing other modifiable risk factors to further decrease the incidence of SSI. Future multicenter, prospective studies are needed to confirm these findings and strengthen evidence-based guidelines for abdominoplasty in Qatar.

## 5. CONCLUSION

In this retrospective cohort, the overall incidence of SSI was lower than that reported in international series, although it was higher in combined procedures compared with simple abdominoplasties. Elevated postoperative glucose levels and suboptimal adherence to antibiotic prophylaxis protocols emerged as key areas for improvement.

## FUNDING

The author(s) received no financial support for the research, authorship, and/or publication of this article.

## ACKNOWLEDGEMENTS

The authors would like to thank the nursing staff from the surgical department for their support.

## CONFLICT OF INTEREST

The author(s) declare that there is no conflict of interest.

## ETHICAL APPROVAL

The study was approved by the Institutional Review Board and the Medical Research Center (MRC-01-25-1039; date: January 1, 2025), Doha (Qatar).

## AUTHORS CONTRIBUTIONS

HGG and ORER: Study design. HGG, ENC, and ORER: Data acquisition. HGG, YLR, ORER, GMG, YHJ, YCC, ENC, OIEM, ILLG, YCF, TMFH, and FGG: Data analysis, critical review, and major scientific input. HGG: Manuscript writing.

## DISCLOSURE OF AI USE

Perplexity AI was utilized exclusively for English language editing and reference formatting corrections in this manuscript. No other aspects of the article’s content, analysis, or authorship were generated or modified using AI tools.

## Figures and Tables

**Table 1.** Potential factors related to surgical site infections after abdominoplasty.

Factors	Total	Surgical site infection		*P* value
		Yes	No	
		No. (%)	No. (%)	
Age (years)	40 (9.8)	44.1 (11.1)*	40.3 (9.7)*	0.18
**Sex**				
Male	47	4 (8.5)	43 (91.5)	0.47
Female	276	13 (4.7)	263 (95.3)	
**Smoking**				
Never smoker	270	14 (5.2)	256 (94.8)	0.90
Former smoker	4	0 (0.0)	4 (100)	
Current smoker	39	2 (5.1)	37 (94.9)	
Diabetes mellitus	45	5 (11.1)	40 (88.9)	0.12
**Nutritional status according to BMI**				
Underweight (<18.5 kg/m^2^)	1	0 (0.0)	1 (100)	0.26
Normal (18.5–24.9 kg/m^2^)	41	0 (0.0)	41 (100)	
Overweight (≥25.0 kg/m^2^)	164	12 (7.3)	152 (92.7)	
Obesity (≥30.0 kg/m^2^)	117	5 (4.3)	112 (95.7)	
**Hair removal**				
Clipping	165	9 (5.5)	156 (94.5)	0.73
Shaving	118	7 (5.9)	111 (94.1)	
Depilatory cream or laser	38	1 (2.6)	37 (97.4)	
**Type of abdominoplasty**				
Simple	275	12 (4.3)	263 (95.6)	0.15
Combined	48	5 (10.6)	43 (89.6)	
Time from preoperative clinic to admission (days)	43.5 (47.2)	34.6 (30.9)*	43.9 (47.8)*	0.68
Procedure duration (min)	170 (38.7)	163.5 (25.8)*	170.4 (39.3)*	0.45
**Risk score**				
0	236	12 (5.1)	224 (94.9)	0.82
1	82	5 (6.1)	77 (93.9)	
2	5	0 (0.0)	5 (100)	
**ASA Score**				
I	60	3 (5.0)	57 (95.0)	0.97
II	248	13 (5.2)	235 (94.8)	
III	15	1 (6.7)	14 (93.3)	
CHG shower performed the night before the procedure	321	17 (5.3)	304 (94.7)	1.00
CHG shower performed the day of the procedure	321	17 (5.3)	304 (94.7)	1.00
**Glycemia (mmol/L)**				
Before admission	5.4 (1.2)	5.8 (1.7)*	5.4 (1.2)*	0.26
Upon admission	6.0 (1.6)	7.3 (2.1)*	5.9 (1.6)*	0.08
Preoperatory	5.7 (1.3)	6.6 (2.2)*	5.7 (1.2)*	0.35
Post operatory	8.2 (2.2)	10.3 (3.2)*	8.0 (2.0)*	0.04
The day after the procedure	6.6 (1.3)	7.4 (1.8)*	6.5 (1.2)*	0.19
Timing of antibiotic prophylaxis (min)	33.2 (8.5)	35.2 (9.3)*	33.1 (8.4)*	0.56
**Compliance with antibiotic prophylaxis**				
Compliance with timing	321	17 (5.3)	304 (94.7)	1.00
Compliance with selection	323	17 (5.3)	306 (94.7)	---
Compliance with the dose	307	16 (5.2)	291 (94.8)	1.00
Compliance with discontinuation	255	13 (5.1)	242 (94.9)	1.00
Booster doses administer	189	9 (4.8)	180 (95.2)	0.82
Time between loading and booster dose (min)	180.3 (12.7)	176.7 (10.3)*	180.4 (12.8)*	0.40
Postoperative antibiotics postoperatively (días)	1.3 (0.8)	1.3 (0.8)*	1.3 (0.9)*	0.76
Post-discharge antibiotics were prescribed	31	3 (9.7)	28 (90.3)	0.46
Antibiotic prescribed during the first 30 days after discharge**	59	12 (70.5)	47 (15.4)	0.00

Data presented as No (%) unless specified.

CHG, chlorhexidine gluconate.

*Mean (standard deviation).

**Include only the patients for whom no antibiotics were prescribed at discharge.

**Table 2.** Annual antibiotic consumption (DDD per 100 patients) and cost (QAR per 100 patients) according to the presence of surgical site infection in abdominoplasty procedures.

	Surgical site infection								
	Yes			No			Total		
**Antibiotic consumption**	**Procedures**	**DDD**	**DDD/100**	**Procedures**	**DDD**	**DDD/100**	**Procedures**	**DDD**	**DDD/100**
2022				49	188.7	385.1	49	188.7	385.1
2023	4	8.5	212.5	54	149.0	275.9	58	157.5	271.6
2024	5	9.3	186.0	99	201.2	203.2	104	210.5	202.4
2025	8	42.7	533.8	104	319.3	307.0	112	362.0	323.2
Total	17	60.5	355.9	306	858.2	280.5	323	918.7	284.4
**Antibiotic cost**	**Procedures**	**Cost**	**QR/100**	**Procedures**	**Cost**	**QR/100**	**Procedures**	**Cost**	**QR/100**
2022				49	1202.1	2453.3	49	1202.1	2453.3
2023	4	202.2	5055.0	54	997.6	1847.4	58	1199.8	2068.6
2024	5	84.2	1684.0	99	1872.3	1891.2	104	1956.5	1881.3
2025	8	190.9	2386.3	104	2598.7	2498.8	112	2789.6	2490.7
Total	17	477.3	2807.6	306	6670.7	2180.0	323	7148.0	2213.0

DDD, daily defined doses; QR, Qatari Riyals. Only antibiotics prescribed (for prophylaxis, postoperative use, and at discharge) during the initial admission for the surgical procedure were recorded. *P* value for consumption is 0.45, *P* value for cost is 0.72.
